# Esophagogastroduodenoscopy Quality Improvement and the Role of Topical Antiperistaltic Agents: A Systematic Review and Meta-Analysis

**DOI:** 10.7759/cureus.73855

**Published:** 2024-11-17

**Authors:** Eoghan Burke, Patricia Harkins, Mayilone Arumugasamy

**Affiliations:** 1 Surgery, Royal College of Surgeons in Ireland (RCSI), Dublin, IRL; 2 Medicine, Royal College of Physicians of Ireland (RCPI), Dublin, IRL

**Keywords:** antiperistaltic, esophagogastroduodenoscopy, gastric cancer, premalignant lesions, quality improvement

## Abstract

Gastric cancer (GC) represents one of the most lethal forms of cancer. When identified at an early stage, conventional treatment can be curative. The key to identifying GC at an early stage is high-quality esophagogastroduodenoscopy (EGD).

This has led to an increased focus on quality standards in EGD to improve the detection rates of early GC and its premalignant lesions (PMLs), such as atrophic gastritis. In Asia, the routine use of antiperistaltic (antispasmodic) agents is advocated to improve the quality of mucosal visualization during diagnostic EGD. The rationale is that the cessation of peristalsis should yield a more stable intragastric visual field to enhance the detection of early GC.

Hyoscine and glucagon are commonly used as antiperistaltic agents. Both, however, must be given either intravenously or via intramuscular injection. They both also have potentially serious systemic side effects, which can limit their routine use, particularly in the elderly or co-morbid patients.

As a result of these side effects, there is growing interest in using peppermint oil or L-menthol topically as anti-peristaltic agents. As these agents are applied topically (either via direct spraying during the EGD or consumed as a premedication before the EGD), they are associated with fewer adverse events than systemically applied agents.

This study aimed to synthesize, for the first time, the available data on the use of topically applied anti-peristaltic agents to decrease or stop peristalsis during diagnostic EGDs. This study is a systematic review and meta-analysis of published randomized controlled trials. Its reporting is per the Preferred Reporting Items for Systematic Reviews and Meta-Analyses (PRISMA) statement. This study was registered prospectively with the PROSPERO register, registration number CRD42024601488.

This systematic review and meta-analysis encompassed five high-quality randomized controlled trials with a low risk of bias. All included studies were conducted in Asian countries between 2011 and 2022. They comprised 538 patients, with a mean age of 62.7. Four of the included studies look at topically applied L-menthol at 160 mg, while one study looked at the role of 160 mg of phloroglucinol administered as a 20 mL oral premedication liquid 15 minutes before the EGD. All included trials involved diagnostic EGDs and reported their primary outcomes using the same scoring systems.

The primary outcome of interest for this study was the efficacy of the antiperistaltic agents at stopping peristalsis for the duration of a diagnostic EGD. We found an odds ratio of 4.22 with a 95% confidence interval ranging from 2.47 to 7.21, favoring the antiperistaltic agents in terms of attaining a peristalsis score of 1 after administration.

This systematic review and meta-analysis represent the most up-to-date review on topical antiperistaltic agents during diagnostic EGD. We found that topical antiperistaltic agents effectively decrease or stop peristalsis during an EGD, and these effects persist for the duration of the EGD. Larger-scale studies will be required to determine whether their routine use translates into increased detection rates of early GC and its PMLs.

## Introduction and background

Gastric cancer (GC) represents one of the most lethal forms of cancer, with a five-year overall survival rate of around 20% [1]. There are, however, significant variations in survival rates between countries. Japan and South Korea have some of the highest incidence rates of GC, but their survival rates are superior to those of Western countries such as Ireland and the UK, which have lower incidence rates [2]. This is likely because they identify GC at earlier stages [3]. The missed GC rate, defined as GC diagnosed within three years of an esophagogastroduodenoscopy (EGD), globally may be as high as 25%, with studies from the UK suggesting rates of 10% [4]. When identified at an early stage, conventional treatment can be curative. The key to identifying GC at an early stage is using high-quality EGD.

As a result, there has been increased interest in quality standards in EGD. Guidelines in Asia advocate the routine use of antiperistaltic (antispasmodic) agents to improve the quality of mucosal visualization during diagnostic EGD [5]. The rationale is that the cessation of peristalsis should yield a more stable intragastric visual field to enhance the detection of early GC. Early GC and premalignant lesions (PMLs) represent subtle mucosal changes that are difficult to identify and can be obscured by gastric folds [6]. Peristalsis can further impair their early detection. Providing a stable intragastric visual field also holds promise for an increased role of artificial intelligence programs in lesion detection [7].

The Asian guidelines support using hyoscine and glucagon as antiperistaltic agents. Both, however, must be given either intravenously or via intramuscular injection. They both also have potentially serious systemic side effects, which can limit their routine use, particularly in the elderly or co-morbid patients. This is particularly interesting as the global population ages, and the proportion of elderly patients undergoing EGD is also increasing [8].

Hyoscine is an anticholinergic agent with side effects, including narrow-angle glaucoma, arrhythmias, and urinary retention. Glucagon is a positive inotrope that can cause hypertension, hyperglycemia, and delayed hypoglycemia [9].

Acknowledging these side effects, the Asian guidelines also advocate using peppermint oil or L-menthol topically as anti-peristaltic agents. As these agents are applied topically (either via direct spraying during the EGD or consumed as a premedication before the EGD), they are associated with fewer adverse events than systemically applied agents.

Peppermint oil's active component is L-menthol. It is commonly used as a food preservative, so its safety profile is well established. Peppermint oil and L-menthol have also been extensively studied in animal models to elucidate their anti-peristaltic effects. When applied directly to the gastric mucosa, the anti-peristaltic effects are exerted via calcium channel-blocking mechanisms on the gastric smooth muscle [10].

Phloroglucinol is a phenol derivative shown to exert anti-peristaltic effects via the selective inhibition of smooth muscle contraction independent of the cholinergic pathway [11]. Like peppermint oil and its extract L-menthol, phloroglucinol can be applied directly onto the gastric mucosa during EGD or taken as a premedication before the EGD. Similarly, due to its topical application, its side effect profile is thought to be less than that of the traditionally used intravenous or intramuscular anti-peristaltic agents.

The Asian guidelines for quality standards during EGD are unique in advocating for the routine use of anti-peristaltic agents during diagnostic EGD. Neither the British Society of Gastroenterology [12], the European Society of Gastrointestinal Endoscopy [13], nor the American Gastroenterological Association [14] guidelines/position papers for quality standards in EGD currently mention the role of anti-peristaltic agents.

In light of this, we conducted the most up-to-date systematic review and meta-analysis on topically applied anti-peristaltic agents' role in diagnostic EGD to inform future guidelines.

## Review

Study aims

This study aimed to synthesize, for the first time, the available data on the use of topically applied anti-peristaltic agents to decrease or stop peristalsis during diagnostic EGDs.

The specific objectives of this systematic review and meta-analysis were as follows: 1) to assess the efficacy of topically applied anti-peristaltic agents (either sprayed during the endoscopy directly onto the antrum of the stomach or taken pre-endoscopy as a premedication drink) in decreasing or stopping gastric peristalsis; 2) to determine the optimal agent, dose, administration method, and timing; 3) to assess the duration of the effect after a single application of the agent in terms of maintaining the degree of anti-peristaltic effect; and 4) to determine the side effect profile of topically applied anti-peristaltic agents.

To better inform the development of the search strategy, the PICO model was used to define the search criteria (Table 1).

**Table 1:** PICO model used to construct the search strategy.

P	I	C	O
Population	Intervention	Comparator	Outcome
Adult patients undergoing elective non-therapeutic EGD	Topically applied anti-peristaltic agent. Either sprayed down the endoscope or taken as a premedication prior to the EGD	Placebo	Effect on gastric peristalsis as determined by a previously validated peristalsis score

Methods

Study Design

This study is a systematic review and meta-analysis of published randomized controlled trials assessing the role of topically applied anti-peristaltic agents in decreasing or stopping gastric peristalsis during diagnostic EGDs. Its reporting is per the Preferred Reporting Items for Systematic Reviews and Meta-Analyses (PRISMA) statement [15]. This study was registered prospectively with the PROSPERO register, registration number CRD42024601488.

Inclusion and Exclusion Criteria

This study included randomized controlled trials involving adult patients undergoing diagnostic, non-therapeutic EGDs. We included studies that assessed a topically applied antiperistaltic agent versus placebo in terms of decreasing or stopping the degree of peristalsis during a diagnostic EGD. To allow for a robust meta-analysis, we only included studies that reported their primary outcomes using a validated peristalsis scoring system.

We excluded studies that were not randomized controlled trials, trials involving pediatric patients, and trials assessing the efficacy of anti-peristaltic agents during therapeutic EGDs.

Search Strategy

A thorough search strategy was developed in consultation with a medical librarian. The search string used Medical Subject Headings (MeSH) terms and keywords for antispasmodics, antiperistaltic agents, and EGDs. To avoid excluding relevant studies, the search string was not designed to exclude systemically applied antiperistaltic agents. The final search string utilized was: (Antiperistaltic OR antispasmodic) AND (OGD OR EGD OR Oesophagogastroduodenoscopy OR esophagogastroduodenoscopy). This search string was applied and adapted as necessary to the following bibliographic databases: PubMed, EMBASE, and the Cochrane Central Register of Controlled Trials (CENTRAL). All databases were searched from inception up to July 20, 2024, and no language restrictions were imposed.

Study Selection

The studies yielded by applying this search string to the bibliographic databases were then uploaded to the systematic review management software package [16]. Duplicates were automatically identified and removed as required after an independent review by two of the authors. After removing the identified duplicates, two authors independently screened the remaining titles and abstracts. Abstracts that met the inclusion criteria were selected, and any conflicts about a study's inclusion were resolved by consensus. The resulting studies were then reviewed in full. Again, any disputes about the eligibility of a study for inclusion in qualitative and quantitative analyses were resolved initially by consensus, and if needed, a third author would decide. To ensure no relevant studies were omitted, hand-searching of the references of the included studies was done alongside a citation search using Google Scholar [17].

Data Extraction

Two authors independently extracted data using a predefined data extraction form developed using Microsoft Excel. The data extracted included the study title, authors, year of publication, country of origin, the active agent used, method of administration, dose, and timing of administration, outcome measures using a predefined peristalsis scoring system, adverse events, and patient demographic data.

Risk of Bias Assessment

The Cochrane Collaboration risk of bias tool was used to assess each study's risk of bias objectively. Two authors performed this assessment independently, and a third author resolved any disagreements as needed. The results are displayed graphically using RevMan Software from the Cochrane Collaboration.

Summary Measures and Synthesis of Results

An objective scoring system to report the degree of peristalsis is essential to assessing the efficacy of topically applied antiperistaltic agents in decreasing or stopping intra-gastric peristalsis. After identifying the included studies, the authors conducted a qualitative review to determine the most widely used peristalsis scoring system. Following this review, the peristalsis scoring system developed by Hiki et al. [18] was found to be the most commonly used. It describes five degrees of peristalsis as outlined in Table 2. Hiki et al. suggest that grades 1 and 2 would allow for a high-quality EGD.

**Table 2:** Classification of Gastric Peristalsis Scoring System by Hiki et al. Reference: [18]

Grade of peristalsis	Description of findings
1	No peristalsis - no or very weak gating movement of the pyloric ring is observed, but the movement does not show strong contraction.
2	Mild peristalsis - a circular peristaltic wave is formed in the antrum but disappears without reaching the pyloric ring, or circular contraction temporarily occurs immediately before the pyloric ring.
3	Moderate peristalsis - a pronounced peristaltic wave is formed and reaches the pyloric ring.
4	Vigorous peristalsis - peristaltic wave is deep and pronounced and proceeds, strangulating the antrum.
5	Markedly vigorous peristalsis - peristaltic wave is even deeper and more pronounced, and the entire antrum appears severely strangled.

To further augment the assessment of the efficacy of antiperistaltic agents, Hiki et al. also developed an easy intra-gastric visualization scoring system. This system is also widely used. It breaks the ease of intra-gastric visualization into four grades, as described in Table 3.

**Table 3:** Ease of intragastric observation scoring system developed by Hiki et al. Reference: [18]

Grade	Description
Very easy	No peristalsis was noted and no interruption of observation
Easy	Mild peristalsis noted but no interruption or interference with observation
Slightly difficult	Peristalsis was noted and interfered with observation slightly
Difficult	Marked peristalsis was noted and made observation difficult

This systematic review and meta-analysis included only trials that reported their outcomes using these scoring systems.

Results

Study Selection

**Figure 1:**
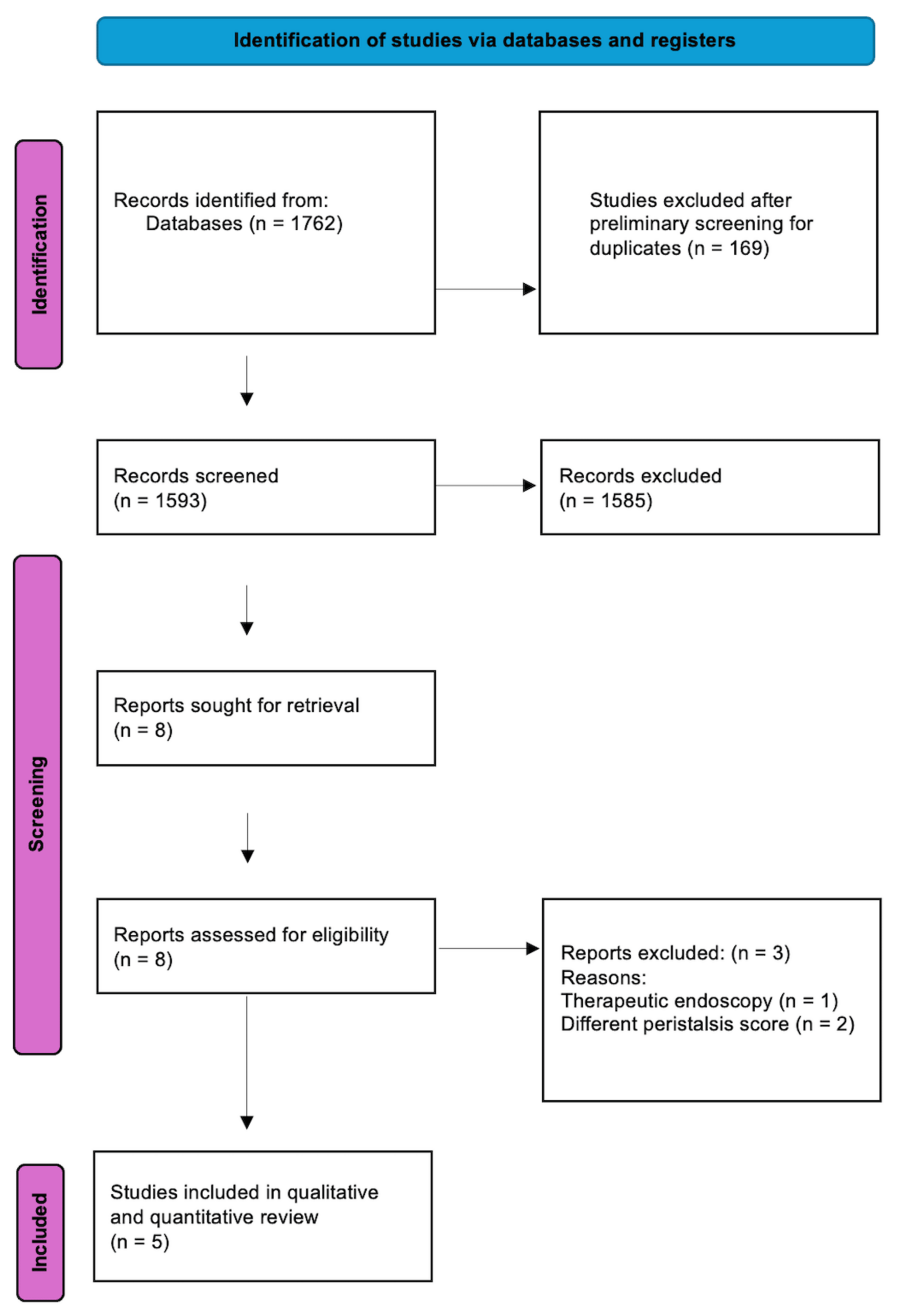
PRISMA flowchart depicting the study selection process for this systematic review and meta-analysis. PRISMA: Preferred Reporting Items for Systematic Reviews and Meta-Analyses

After applying the search string to the bibliographic databases, 1762 articles were identified. The titles and abstracts were transferred to the systematic review management software. Duplicates were determined by the software and were rationalized independently by two of the authors. Following the removal of the duplicates, 1593 articles remained to be screened. Two authors independently screened the title and abstract; any disagreement about the eligibility of an article for inclusion was resolved by consensus, and if needed, a third author would decide. After completing this phase, eight articles were identified for a full review. Following this, three were excluded for reasons. This resulted in five articles being identified as suitable for inclusion in qualitative and quantitative reviews. The PRISMA flow diagram depicting this study selection process is shown in Figure 1.

Study Characteristics

As outlined above, five studies were selected for inclusion in qualitative and quantitative reviews [18-22]. The characteristics of the included studies are described in Tables 4-5. The included studies were conducted between 2011 and 2022. All studies were conducted in Asian countries. The studies comprised four randomized phase 3 trials and one phase 2 trial. All studies were double-blinded and randomized using computer-generated random numbers, and a placebo was used as a control arm. In total, 538 patients were included, with a mean age of 62.7 and an average of 58.6% male participants.

**Table 4:** Characteristics of included studies: year of study, country of origin, study design and phase, degree of blinding, method of randomization, and control details. RCT: randomized controlled trial

Study ID	Year	Country	Study design	Blinding	Randomization method	Control
Yang et al. [20]	2022	Taiwan	RCT-Phase 3	Double-blinded	Computer generated	Placebo
Jung et al. [19]	2021	Korea	RCT-Phase 3	Double-blinded	Computer generated	Placebo
Meng et al. [22]	2021	China	RCT-Phase 3	Double-blinded	Computer generated	Placebo
Hiki et al. [18]	2011	Japan	RCT-Phase 3	Double-blinded	Computer generated	Placebo
Kaminishi et al. [21]	2012	Japan	RCT-Phase 2	Double-blinded	Computer generated	Placebo

**Table 5:** Characteristics of included studies, number of patients, mean age, percentage of male patients, and breakdown of those receiving active versus placebo.

Study ID	Total number of patients	Mean age	% male	Number receiving antiperistaltic	Number receiving placebo
Yang et al. [20]	52	82.1	67	26	26
Jung et al. [19]	142	59	52	71	71
Meng et al. [22]	220	51.5	51	109	111
Hiki et al. [18]	87	63.5	62	45	42
Kaminishi et al. [21]	37	57.4	61	19	18

The technical aspects of the endoscopic procedures of each of the five included studies are described in Table 6. Four of the included studies used L-Menthol at 160 mg, which was applied topically via spraying through the endoscope’s working channel. In one study, Jung et al. [19] used phloroglucinol 160 mg, which was taken as a 20 ml premedication solution 15 minutes before the EGD. All of the included studies used the same peristalsis scoring system developed by Hiki et al. [18] and were performed on un-sedated patients. The average timing from applying the topical antiperistaltic agent to acquiring the initial peristalsis scores varied from a low of 60 seconds, Yang et al. [20], to a high of 15 minutes, Jung et al. [19], accounting for the differing application methods. The average duration of EGD also varied among the studies from a low of 317 seconds, Jung et al. [19], to a high of 606 seconds, Yang et al. [20], with Kaminishi et al. [21] not reporting their average endoscopy time.

**Table 6:** Technical aspects of the endoscopic procedures performed in each study.

Study ID	Peristalsis scoring system	Indication, sedation	Active used, dose, and method of administration	Average timing of results acquisition post-treatment	Average duration of esophagogastroduodenoscopy
Yang et al. [20]	Hiki et al. system	Diagnostic, unsedated	20 mL of 0.8% preparation (160 mg L-Menthol), sprayed via endoscope	60 seconds	606 seconds
Jung et al. [19]	Hiki et al. system	Diagnostic, unsedated	Phloroglucinol 160 mg in 20 ml solution taken orally as a premedication - 15 minutes before OGD	15 minutes	317 seconds
Meng et al. [22]	Hiki et al. system	Diagnostic, unsedated	20 mL of 0.8% preparation (160 mg L-Menthol), sprayed via endoscope	90 to 135 seconds	380 seconds
Hiki et al. [18]	Hiki et al. system	Diagnostic, unsedated	20 mL of 0.8% preparation (160 mg L-Menthol), sprayed via endoscope	90 to 135 seconds	392.25 seconds
Kaminishi et al. [21]	Hiki et al. system	Diagnostic, unsedated	20 mL of 0.8% preparation (160 mg L-Menthol), sprayed via endoscope	105 seconds	Not reported

Risk of Bias Assessment

**Figure 2:**
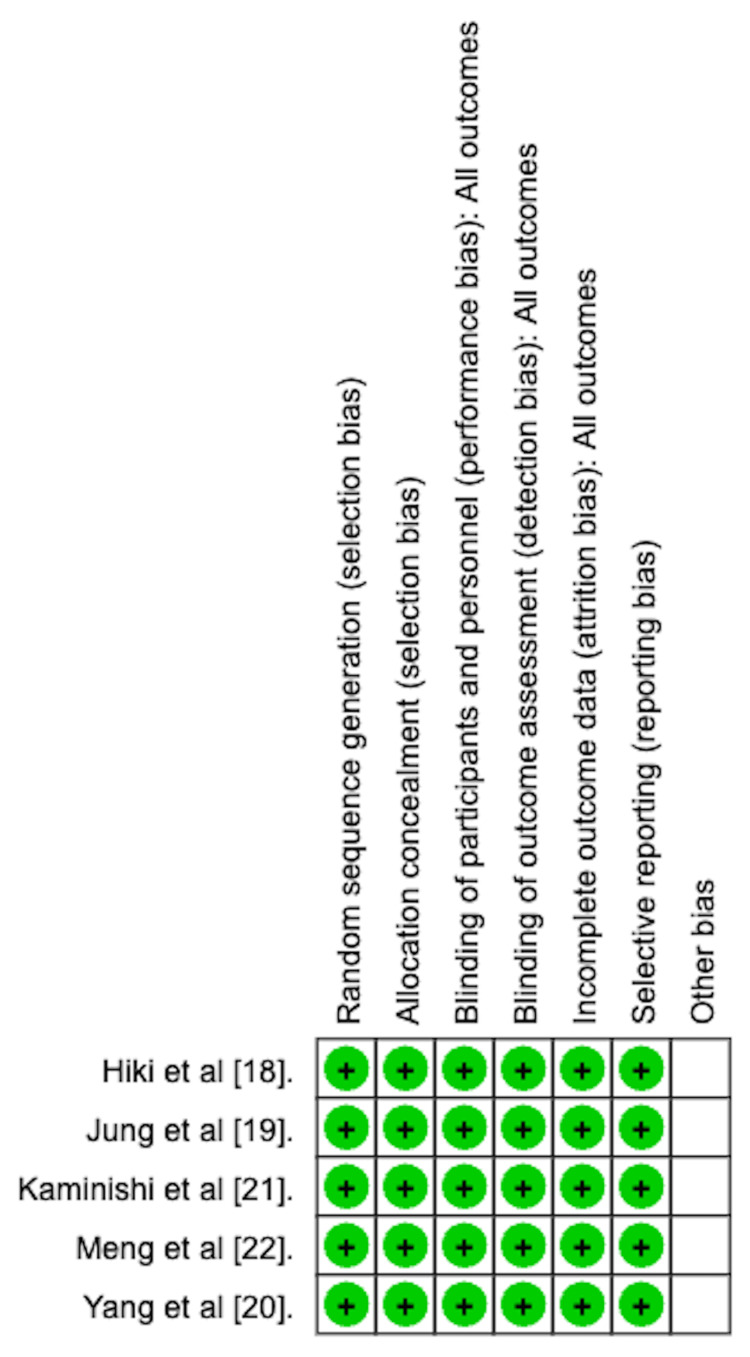
Risk of bias assessment using the Cochrane Collaborations Risk of Bias tool.

Two of the authors independently assessed each study’s risk of bias using the Cochrane Collaboration's Risk of Bias tool. Any conflicts were resolved by consensus. Each study was evaluated for the following biases: selection bias, performance bias, detection bias, attrition bias, and reporting bias. The results are depicted graphically in Figure 2. The included studies were found to have a low risk of biases.

As depicted in Table 4, all included studies were randomized controlled trials that used a computer-generated randomization technique. They were similarly double-blinded and used a placebo as a control arm.

Further steps were also taken to reduce bias. This included the use of Endoscopic Video Evaluation Committees (EVEC). These committees were established to report the primary outcomes. These committees comprised board-certified endoscopists not directly involved with the respective studies. The EVEC was presented with the videos of each endoscopy and reported the peristalsis scores and ease of intra-gastric evaluation scores. Each of the included studies utilized an EVEC.

Synthesis of Results for Meta-Analysis

All of the included studies were deemed suitable for inclusion in the meta-analysis. They all utilized the same peristalsis scoring system and ease of intra-gastric visualization system developed by Hiki et al. [18].

Ease of Intragastric Visualization

**Figure 3:**
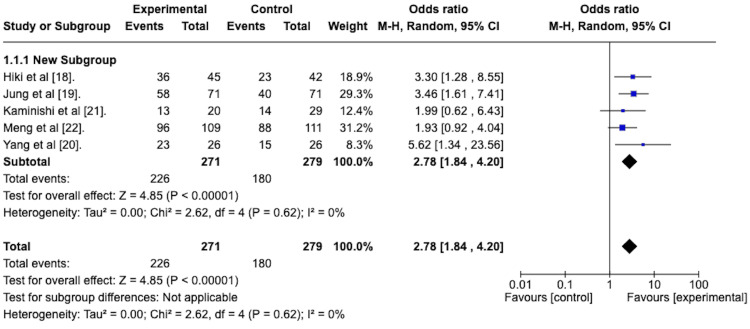
Forest plot depicting the odds ratio of the antiperistaltic agent versus placebo in attaining a score of “very easy” or “easy” on the ease of intragastric visualization scoring system.

All five of the included studies assessed the effect of topically applied antiperistaltic agents on the ease of intragastric visualization compared to placebo using the scoring system developed by Hiki et al. [18]. To determine the odds ratio of the antiperistaltic agent attaining a score of “very easy” or “easy” on the ease of intragastric visualization score, a random-effects meta-analysis was performed. Results are presented graphically as a forest plot in Figure 3. This reveals an odds ratio of 2.78 with a 95% confidence interval ranging from 1.84 to 4.20 in favor of the antiperistaltic agent. The overall effect size, as depicted via the Z-statistic, was 4.85 and was statistically significant with a p-value of less than 0.00001. The studies were homogenous concerning this outcome, as evidenced by the I^2 ^value of 0%.

Grade 1 Peristalsis Post-treatment

As described previously, all of the included studies assessed the efficacy of the topically applied antiperistaltic agent in attaining a score of 1 on the peristalsis scoring system at two time points. The first point is after the agent's application (Table 6) and then again at the end of the EGD. Grade 1 peristalsis indicated the complete absence of peristalsis.

**Figure 4:**
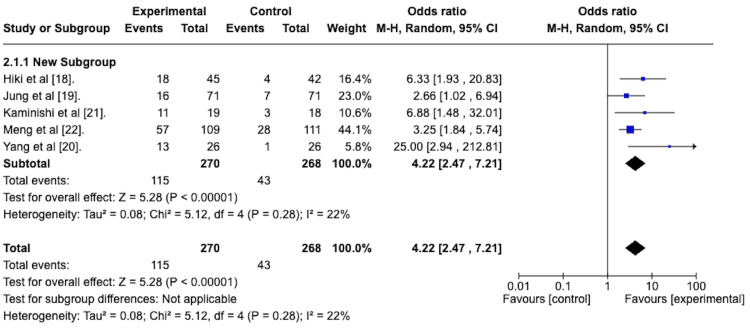
Forest plot depicting the odds ratio of the antiperistaltic agents versus placebo in attaining a peristalsis score of 1 after administration of the agent.

A random-effects meta-analysis was performed to assess the efficacy of the topically applied antiperistaltic agents in attaining a peristalsis score of 1. The odds ratio of the antiperistaltic agent attaining a score of 1 versus the placebo was 4.22, with a 95% confidence interval ranging from 2.47 to 7.21 in favor of the antiperistaltic agent. This is depicted in the forest plot in Figure 4. The Z statistic for the overall effect size was 5.28 and was statistically significant with a p-value of less than 0.00001. The studies were homogenous concerning this outcome, with an I^2^ statistic of 22%.

Peristalsis Grade 1 at the End of EGD

**Figure 5:**
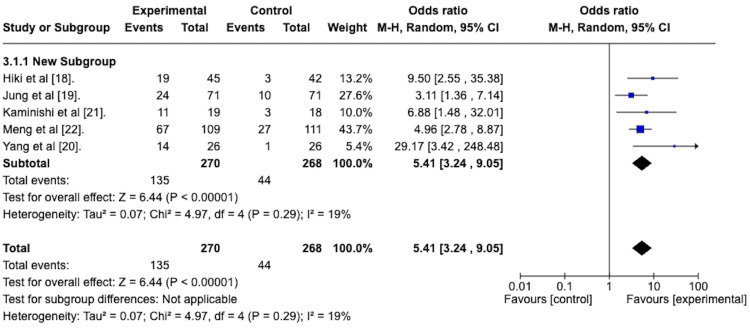
Forest plot depicting the odds ratio of the peristaltic agents versus placebo in attaining a peristalsis score of 1 at the end of the esophagogastroduodenoscopy.

The efficacy of the antiperistaltic agents in attaining a peristalsis score of 1 was also assessed at the end of the EGD, indicative of the persistence of the antiperistaltic effect after a single administration of the agent. A random effects model revealed an odds ratio of 5.41 with a 95% confidence interval from 3.24 to 9.05 in favor of the antiperistaltic agent (Figure 5). The Z statistic for overall effect size was 6.44 and was statistically significant with a p-value of less than 0.00001. The studies were homogenous concerning this outcome, with an I^2^ value of 19%.

Peristalsis Grade 1 or 2 Post-treatment

**Figure 6:**
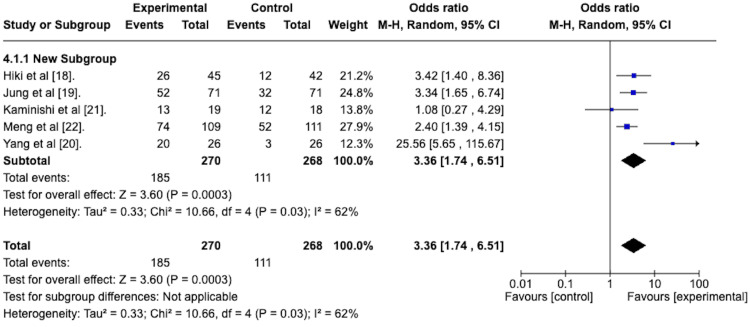
Forest plot depicting the odds ratio of the antiperistaltic agents versus placebo in attaining a peristalsis score of 1 or 2 following the administration of the agent.

As per the peristalsis scoring system described by Hiki et al. [18], a score of 1 indicates the absence of peristalsis, while a score of 2 indicates the presence of mild peristalsis. A high-quality EGD can be performed with scores of 1 or 2. As such, a random effects model was used to determine the odds ratio of attaining a score of 1 or 2 between the antiperistaltic agent and placebo. The resulting forest plot is depicted in Figure 6. This reveals an odds ratio of 3.36 with a 95% confidence interval ranging from 1.74 to 6.51 in favor of the antiperistaltic agent. The Z statistic for overall effect size was 3.60 and was statistically significant with a p-value of 0.0003. The studies were heterogeneous concerning this outcome, with an I^2^ value of 62%.

Peristalsis Grade 1 or 2 at the End of EGD

**Figure 7:**
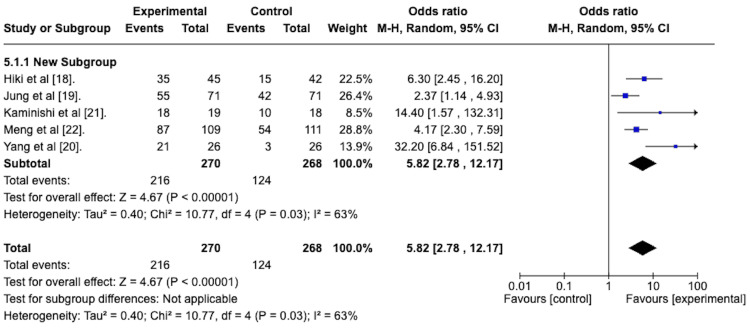
Forest plot depicting the odds ratio of the antiperistaltic agent versus placebo in attaining a peristalsis score of 1 or 2 at the end of the esophagogastroduodenoscopy.

The durability of the antiperistaltic effect was again assessed by determining the odds ratio of attaining a peristalsis score of 1 or 2 at the end of the EGD. A random effects meta-analysis was done to determine this, and the forest plot is depicted in Figure 7. This reveals an odds ratio of 5.82 with a 95% confidence interval from 2.78 to 12.17 in favor of the antiperistaltic agents. The Z-statistic for the overall effect size was 4.67 and was statistically significant with a p-value of less than 0.00001. The studies were heterogeneous concerning this outcome, as evidenced by the I^2^ value of 63%.

Adverse Events

**Figure 8:**
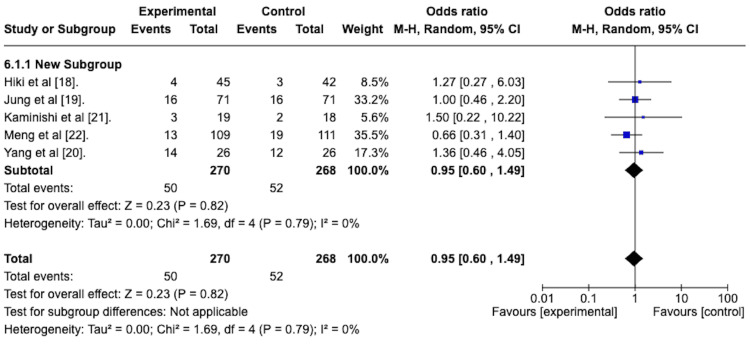
Forest plot depicting the odds ratio of antipersitaltic agents versus placebo in terms of adverse event rates.

With respect to adverse events, Figure 8 represents the forest plot of the random effects meta-analysis to determine the odds ratio of adverse events in the active arm versus the placebo arm of the included studies. This revealed an odds ratio of 0.95 with a 95% confidence interval from 0.60 to 1.49. This suggests a lower rate of adverse events in the antiperistaltic arm. The overall effect size was represented by the Z-statistic of 0.23, which was not statistically significant, and the p-value was 0.82. The studies were homogenous concerning this outcome, as evidenced by the I^2^ value of 0%.

Subgroup Analysis Based on the Method of Administration and Active Agent Used

Four of the included studies assessed the efficacy of topically applied L-menthol. In all four of these studies, the dose and method of administration were the same. About 160 mg of L-menthol was administered as a 20 ml flush down the endoscope during the EGD. Collectively, these studies are referred to below as the spraying technique group. One study assessed the efficacy of phloroglucinol administered as a premedication 20 ml drink administered 15 minutes before the EGD. This study will be referred to below as the premedication group. To guide the optimal active agent and method of administration, we conducted a subgroup analysis to compare these two routes and active agents.

Ease of Intragastric Visualization With Subgroup Analysis

**Figure 9:**
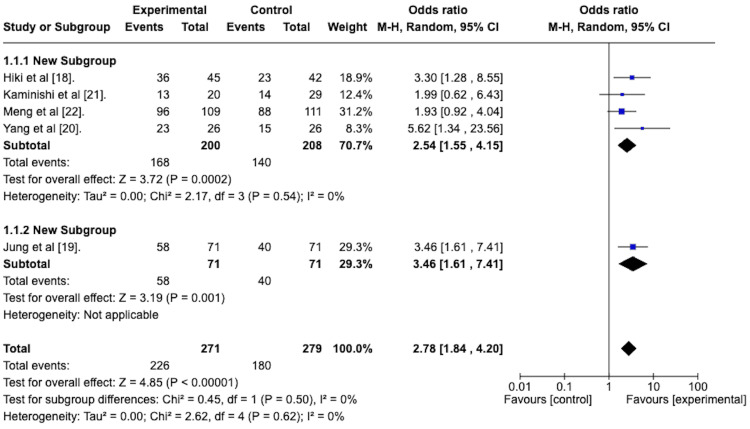
Forest plot depicts the sub-group analysis of the spraying technique group versus the premedication group in terms of the odds ratio of attaining a “very easy” or “easy” score based on the ease of the intragastric visualization scoring system.

Figure 9 depicts the forest plot of the random effects model with subgroup analysis comparing the premedication group to the spraying technique group on ease of intra-gastric visualization.

The odds ratio for the spraying technique group was 2.54, with a 95% confidence interval between 1.55 and 4.15. It was statistically significant, with a p-value of 0.0002.

The odds ratio for the premedication group was 3.46, with a 95% confidence interval from 1.61 to 7.41. It was statistically significant, with a p-value of 0.001.

The test for subgroup differences was not statistically significant, with a p-value of 0.5.

These results reflect the odds ratio of attaining a “very easy” or “easy” score on the ease of intra-gastric visualization score.

Grade 1 Peristalsis Post-treatment With Subgroup Analysis

**Figure 10:**
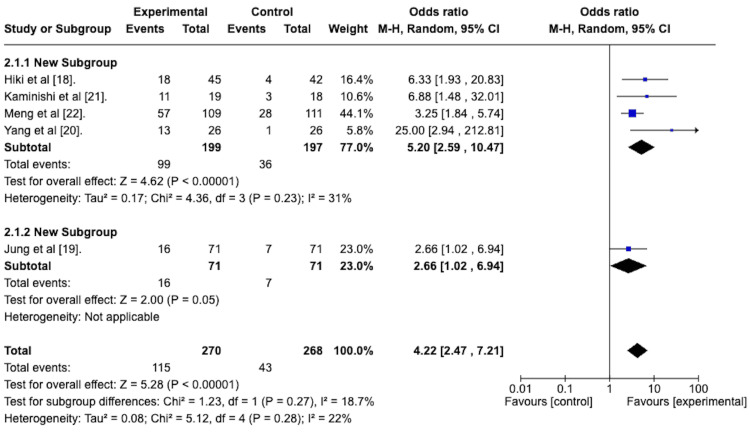
Forest plots depicting the sub-group analysis of the spraying technique group versus the premedication group in terms of the odds ratio of attaining a peristalsis score of 1 following administration of the agent.

A random-effects meta-analysis was conducted with a subgroup analysis to determine the efficacy of the premedication group versus the spraying technique group in attaining a grade 1 peristalsis score after the initial application of the active agent. The forest plot is depicted in Figure 10.

The odds ratio for the spraying technique group was 5.20 with a 95% confidence interval from 2.59 to 10.47 and was statistically significant with a p-value of less than 0.00001.

The odds ratio for the premedication group was 2.66 with a 95% confidence interval from 1.02 to 6.94, which was statistically significant with a p-value of 0.05.

The test for subgroup differences was not statistically significant with a p-value of 0.27.

*Peristalsis Grade 1 at the End of EGD* *With Subgroup Analysis*

**Figure 11:**
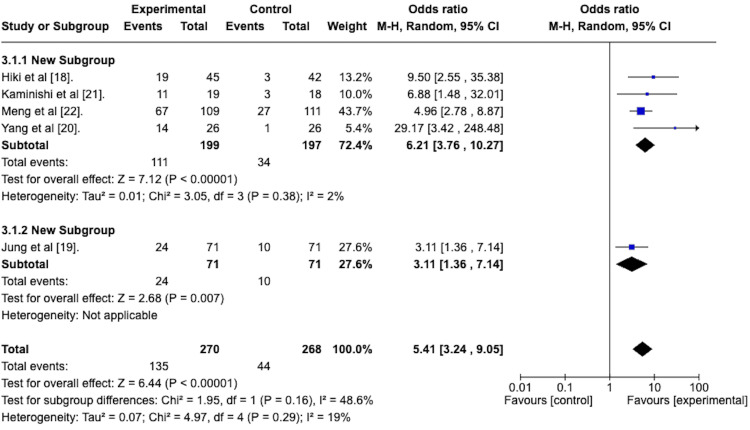
Forest plot depicting the sub-group analysis of the spraying technique group versus the premedication group regarding the odds ratio of attaining a peristalsis score of 1 at the end of the esophagogastroduodenoscopy.

A random effects meta-analysis with a subgroup analysis was performed to determine the efficacy of the premedication group versus the spraying technique group in attaining a grade 1 peristalsis score at the end of the EGD. The forest plot is depicted in Figure 11.

The odds ratio for the spraying technique group was 6.21 with a 95% confidence interval from 3.76 to 10.27 and was statistically significant with a p-value of less than 0.00001.

The odds ratio for the premedication group was 3.11, with a 95% confidence interval from 1.36 to 7.14. It was statistically significant with a p-value of 0.007.

The test for subgroup differences was not statistically significant, with a p-value of 0.16.

Peristalsis Grade 1 or 2 Post-treatment With Subgroup Analysis

**Figure 12:**
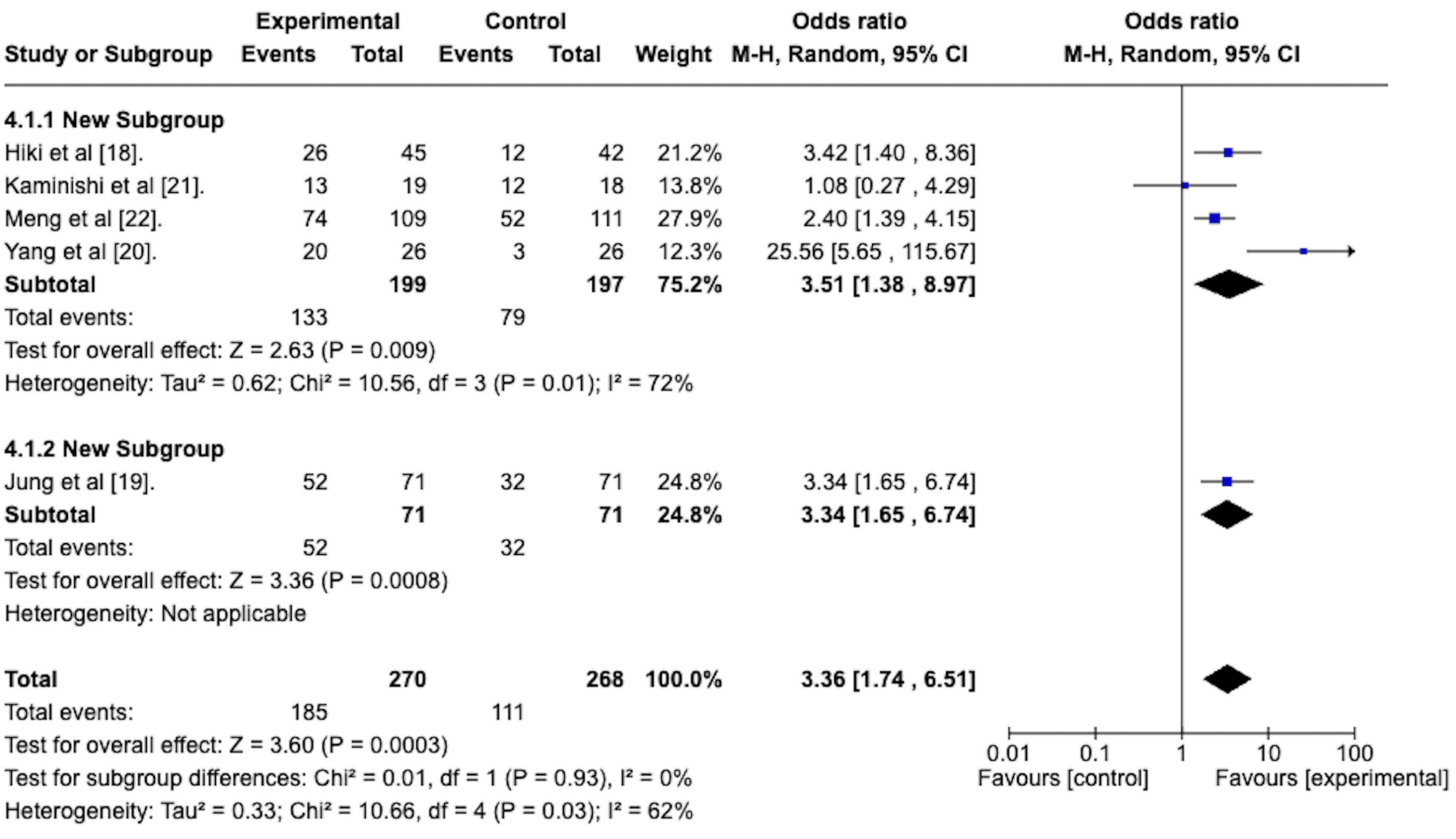
Forest plot depicting the sub-group analysis of the spraying technique group versus the premedication group in terms of the odds ratio of attaining a peristalsis score of 1 or 2 after administration of the agent.

A random effects meta-analysis with subgroup analysis was performed to compare the efficacy of the premedication group versus the spraying technique group in attaining a grade 1 or 2 peristalsis score after the agent was administered. This is depicted in the forest plot in Figure 12.

The odds ratio for the spraying technique group was 3.51, with a 95% confidence interval from 1.38 to 8.97. It was statistically significant, with a p-value of 0.009.

The odds ratio for the premedication group was 3.34, with a 95% confidence interval from 1.65 to 6.74, and was statistically significant, with a p-value of 0.0008.

The test for subgroup differences was not statistically significant, with a p-value of 0.93.

*Peristalsis Grade 1 or 2 at the End of EGD* *With Subgroup Analysis*

**Figure 13:**
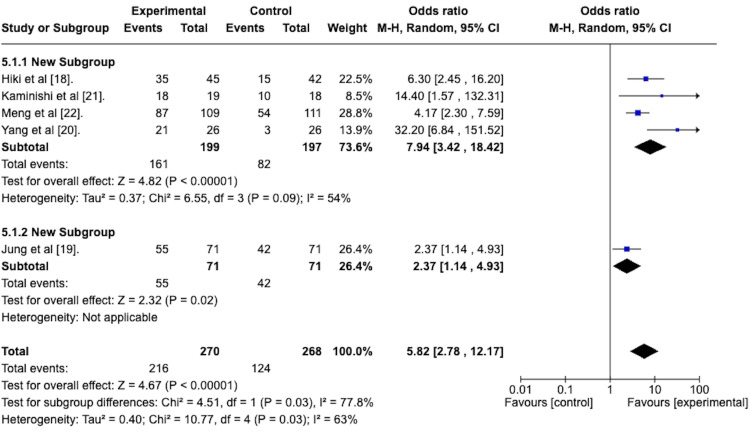
Forest plot depicting the sub-group analysis of the spraying technique group versus the premedication group regarding the odds ratio of attaining a peristalsis score of 1 or 2 at the end of the esophagogastroduodenoscopy.

To assess the efficacy of the spraying technique group compared to the premedication group in attaining a peristalsis grade 1 or 2 score at the end of the EGD. A random effects model with subgroup analysis was performed. The forest plot for this model is depicted in Figure 13.

The odds ratio for the spraying technique group was 7.94 with a 95% confidence interval from 3.42 to 18.42 and was statistically significant with a p-value less than 0.00001.

The odds ratio for the premedication group was 2.37, with a 95% confidence interval from 1.14 to 4.93. It was statistically significant, with a p-value of 0.02.

The test for subgroup differences was statistically significant, with a p-value of 0.03.

Adverse Events

**Figure 14:**
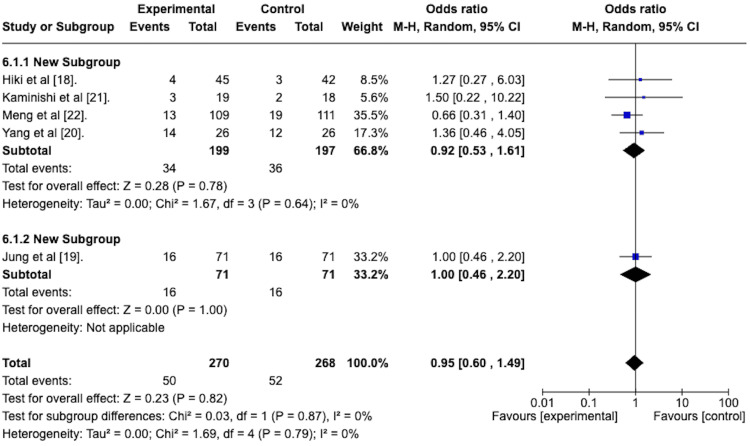
Forest plot depicting the sub-group analysis of the spraying technique group versus the premedication group regarding the odds ratio of adverse events.

A random effects model with subgroup analysis was performed to assess the adverse event rates in the spraying technique group compared to the premedication group. The forest plot is depicted in Figure 14.

The odds ratio for adverse events for the spraying technique group was 0.92, with a 95% confidence interval from 0.53 to 1.61. It was not statistically significant, with a p-value of 0.78.

The odds ratio for adverse events for the premedication group was 1, with a 95% confidence interval of 0.46 to 2.20. This was not statistically significant, with a p-value of 1.

The test for subgroup differences was not statistically significant, with a p-value of 0.87.

Discussion

The most significant predictor of survival in GC is the stage at which the patient is diagnosed [23]. The earlier the GC can be identified, the greater the chance of a cure. Early diagnosis involves using high-quality EGD to identify early GC and its PMLs, such as gastric atrophy. Despite using EGD, the missed GC diagnosis rates - defined as GC diagnosed within three years of an OGD - are as high as 15% in some Western countries [24]. Due to methodological issues in conducting studies into the rate of missed GCs, this figure may be higher.

This missed GC rate has prompted increased interest in improving the quality of EGD. As part of this, there is growing interest in the role of antiperistaltic agents in ensuring high-quality EGD. The rationale is to render the intragastric mucosal field stationary for the duration of the diagnostic EGD. Decreasing or stopping peristalsis should provide the endoscopists with optimal conditions to perform a high-quality EGD. Furthermore, providing a stable intragastric visual field may lay the foundation for an increased role of artificial intelligence platforms in lesion detection [25]. To date, the routine use of antiperistaltic agents has been sparse, and, if used at all, will typically involve the intravenous or intramuscular forms.

As discussed previously, these intravenously and intramuscularly applied agents are associated with potentially significant adverse risks. As such, topically applied agents are growing in popularity in Asian countries. These agents are applied by flushing down the endoscope during the EGD or as a liquid premedication before the EGD. To provide evidence for their routine use in lower-risk Western countries, we have conducted the most up-to-date and detailed systematic review and meta-analysis on topically applied anti-peristaltic agents in diagnostic EGDs.

You et al. (2020) conducted a systematic review and meta-analysis looking at the efficacy of L-menthol in endoscopy in general, both EGD and colonoscopy [26]. This study included both diagnostic and therapeutic endoscopies. Crucially, this study included a therapeutic EGD study that assessed the use of L-menthol during gastric endoscopic mucosal resection procedures. We did not include this study as we were focused on the role of antiperistaltic agents in diagnostic EGDs and wanted to be able to make recommendations about the duration of their effect, acknowledging that a high-quality EGD should take approximately seven minutes [12]. Furthermore, since the publication of You et al.’s study, three further randomized controlled trials have been conducted and have been included in this systematic review and meta-analysis.

This systematic review and meta-analysis encompassed five high-quality randomized controlled trials with a low risk of bias. All included studies were conducted in Asian countries between 2011 and 2022. They comprised 538 patients, with a mean age of 62.7 and an average of 58.6% male participants. Four of the included studies look at topically applied L-menthol at 160 mg, while one study looked at the role of 160 mg of phloroglucinol administered as a 20 mL oral premedication liquid 15 minutes before the EGD. All included trials involved diagnostic EGDs and reported their primary outcomes using the same scoring systems.

There were differences among the studies regarding the timing at which the initial peristalsis scores were taken after the agent was administered (Table 6). Similarly, there were differences concerning the overall duration of the EGD (Table 6).

The primary outcome of interest for this study was the efficacy of the antiperistaltic agents at stopping peristalsis for the duration of a diagnostic EGD. We found an odds ratio of 4.22 with a 95% confidence interval ranging from 2.47 to 7.21, favoring the antiperistaltic agents in terms of attaining a peristalsis score of 1 after administration. This antiperistaltic effect seemed to persist for the duration of the EGDs, as evidenced by the odds ratio of 5.41 with a 95% confidence interval from 3.24 to 9.05 in favor of the antiperistaltic agent for attaining a peristalsis grade of 1 at the end of the EGD.

As previously discussed, a high-quality EGD is possible with peristalsis grades of 1 or 2. We conducted a meta-analysis to assess the efficacy of the antiperistaltic agents in attaining these grades after the initial application of the agent and again at the end of the EGD. After initial application, our study revealed an odds ratio of 3.36 with a 95% confidence interval ranging from 1.74 to 6.51, favoring the antiperistaltic agent. This was statistically significant with a p-value of 0.0003. At the end of the EGD, the odds ratio was 5.82, with a 95% confidence interval of 2.78 to 12.17, favoring the antiperistaltic agents. This, again, was statistically significant with a p-value of less than 0.00001. Thus, the antiperistaltic agents appear effective in attaining peristalsis grades of 1 or 2, and their effect seems to last for the length of the EGD.

This ability to attain peristalsis grades of 1 or 2 also seems to translate into improved intra-gastric visualization based on the ease of intra-gastric visualization scores assessed in this study. We conducted a meta-analysis to evaluate the efficacy of the antiperistaltic agents in attaining a score of “very easy” or “easy” on this system. This revealed an odds ratio of 2.78 with a 95% confidence interval ranging from 1.84 to 4.20 in favor of the antiperistaltic agent. This was statistically significant, with a p-value of less than 0.00001.

The antiperistaltic agents also seemed well tolerated, as evidenced by the odds ratio of 0.95 with a 95% confidence interval from 0.60 to 1.49 (Figure 8). This suggests a lower rate of adverse events in the antiperistaltic arm. However, this finding was not statistically significant, with a p-value of 0.82. It should be noted that, on a detailed review, there were no serious adverse events. The most common adverse event was dry mouth, which was self-limiting.

Our systematic review and meta-analysis focused on all topically applied antiperistaltic agents. Two were identified, namely L-menthol and phloroglucinol. All studies assessing L-menthol applied the same dose via a flush down the endoscope during the EGD. In contrast, the single study assessing phloroglucinol assessed it as a premedication drink taken 15 minutes before the EGD. We conducted a subgroup analysis on the L-menthol (spraying technique) versus the phloroglucinol (premedication group) groups concerning all outcomes (Figures 9-14). We found no statistically significant subgroup differences except the odds ratio of attaining a peristalsis score of 1 or 2 at the end of the EGD. In this case, the L-menthol (spraying technique) was superior. However, whether the spraying technique is superior or just the L-menthol active agent is superior to the phloroglucinol active agent is unclear.

There are limitations to our meta-analysis, which warrants caution when interpreting the results of this research. The primary and secondary outcomes were primarily based on the scoring systems developed by Hiki et al. [18]. Whilst these scoring systems are the most commonly used, and every effort was made to ensure a high degree of intra- and inter-observer agreement, by their very design, they may be susceptible to subjective interpretations. Each study aimed to limit this susceptibility using the Endoscopic Video Evaluation Committees. Similarly, in reporting this systematic review and meta-analysis, we grouped the peristalsis grades 1 and 2 together in an attempt to further mitigate intra- and interobserver differences.

## Conclusions

Early GC and its PMLs can be difficult to identify endoscopically. This systematic review and meta-analysis represent the most up-to-date review on topical antiperistaltic agents during diagnostic EGD. We found that topical antiperistaltic agents effectively decrease or stop peristalsis during an EGD, and these effects persist for the duration of the EGD. More studies are needed to define which method of administration and which agents are superior. Similarly, larger-scale studies will ultimately be required to determine whether the routine use of topically applied antiperistaltic agents during diagnostic EGD translates into increased detection rates of early GC and its PMLs.
